# Evaluation of Hemolysis Risk, Renal Function, and Safety Among Two Pulsed Field Ablation Systems: A Systematic Review and Meta‐Analysis

**DOI:** 10.1002/joa3.70450

**Published:** 2026-08-31

**Authors:** Abdalrahman Al‐Slaimieh, Ibrahim Ghayada, Rand Al‐Huneidy, Mohammad Shahin, Khaled Abukhalaf, Waleed Alomari, Khaled Marwa, Saeed Abdel Kamel, Yahya Almazaraa, Mohammad Hajjiri, Abdallah Al‐Ani

**Affiliations:** ^1^ Department of Electrophysiology Abdali Medical Center Amman Jordan; ^2^ Department of Cardiology Liverpool Heart and Chest Hospital NHS Foundation Trust Liverpool UK; ^3^ School of Medicine The University of Jordan Amman Jordan; ^4^ Department of Renal Surgery University Hospital Birmingham Birmingham UK; ^5^ Epsom and St Helier University Hospitals NHS Trust Sutton UK; ^6^ Department of Internal Medicine University Hospitals Dorset NHS Foundation Trust Bournemouth UK; ^7^ Department of Cardiology Royal Brompton Hospital London UK; ^8^ Division of Vascular and Interventional Radiology Boston Children's Hospital Boston Massachusetts USA

**Keywords:** circular catheter, hemolysis, pentaspline catheter, pulsed field ablation, renal function, safety

## Abstract

This study investigated the hemolysis risk, renal, and safety outcomes between two pulsed field ablation (PFA) systems. A systematic review and meta‐analysis were performed using PubMed, Cochrane, and ScienceDirect databases for all studies comparing circular to pentaspline PFA systems. Pooled markers evaluated included procedural characteristics, haptoglobin, lactate dehydrogenase (LDH), bilirubin, hemoglobin, and creatinine, with glomerular filtration rate (GFR) and safety markers qualitatively discussed. A total of 11 studies were included, comprising 1517 individuals. While differences in procedural, left atrial dwelling, and fluoroscopy times were statistically insignificant between the two PFA systems, the pentaspline system was significantly associated with lower post‐procedural haptoglobin levels (SMD: 0.59; 95% CI: 0.25 to 0.93), higher LDH (SMD: −0.87; 95% CI: −1.49 to −0.253), and higher bilirubin levels (SMD: −0.77; 95% CI: −1.13 to −0.42) compared to circular catheters. No significant differences were observed in total hemoglobin levels (SMD: 0.13; 95% CI: −0.17 to 0.44; *p* = 0.206) or the magnitude of creatinine change (SMD: −0.02; 95% CI: −0.07 to 0.030; *p* = 0.270) between the systems. However, the incidence of acute kidney injury (AKI) was more pronounced in the pentaspline cohort with five documented cases, and more peri/post‐procedural complications were recorded for the pentaspline system than for circular catheters. Pentaspline systems were associated with more pronounced laboratory markers of hemolysis, although clinically significant renal dysfunction remained uncommon and evidence regarding clinical consequences remains limited. Future studies are encouraged to standardize both reporting and data collection procedures to facilitate direct comparisons and tests of validity.

## Graphical abstract

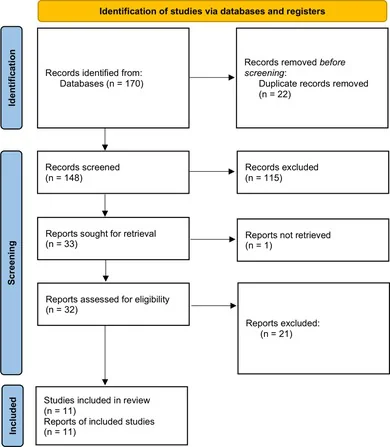

## 1 Introduction

Pulsed Field Ablation (PFA) presents itself as a novel treatment modality for Atrial Fibrillation (AF). To accomplish pulmonary vein isolation, it utilizes a high‐intensity electrical field to increase cardiac myocytes' cell membrane permeability and induce apoptosis [1]. This approach enhances tissue selectivity and causes less injuries to adjacent structures based on pre‐clinical data [2]. Also, clinical data showed decreased rates of major vascular, pericardial, esophageal, and phrenic nerve complications after PFA [3]. Despite its established efficacy and safety profiles, recent studies utilizing PFA as a primary treatment modality demonstrated rare, yet clinically significant, complications such as intravascular hemolysis, coronary arterial spasm, and silent cerebral events and lesions [4, 5, 6].

In vitro studies have shown that erythrocytes are more susceptible to electrical field damage in comparison to cardiac myocytes. More specifically, 1500 V/cm is the amount of energy required to induce cardiac myocytes' apoptosis, whereas 1000 V/cm is enough to induce erythrocytes' hemolysis [7]. These findings were clinically evident in the form of increased serum levels of LDH and bilirubin, and decreased level of haptoglobin post PFA when compared with thermal ablation [8]. Furthermore, this increased incidence of intravascular hemolysis with PFA was associated with a slight increase in the risk of acute kidney injury (AKI) when compared to radiofrequency ablation [9]. The number of peri‐procedure PFA applications and the degree of IV hydration post‐procedurally were found to be associated with the risk of renal insult after PFA [10].

However, the differences in the extent of intravascular hemolysis and subsequent renal insult between different PFA systems were previously investigated only within observational studies. The available secondary syntheses published within the literature do not investigate intra‐system differences in hemolysis risk. Kilic et al. compared the hemolysis and renal safety profiles between PFA and thermal ablation [8], and demonstrated that the earlier increased biomarker‐defined intravascular hemolysis without impacting AKI severity levels. Additionally, Barbosa et al. showed a higher frequency of AKI in PFA compared to radiofrequency ablation irrespective of system [9].

Therefore, we conducted this systematic review and meta‐analysis to further investigate the differences between circular and pentaspline catheters for PFA in terms of the risk and extent of intravascular hemolysis.

## 2 Materials and Methods

### 2.1 Evidence Acquisition

A systematic search of the PubMed/MEDLINE, Cochrane/CENTRAL, and Web of Science databases was conducted from conception to April 2026. Search terms and queries including the following keywords and their derivatives: (“pulsed field ablation” OR “PFA”) AND (“circular catheter” OR “variable loop” OR “PulseSelect (PS)” OR “VariPulse (VP”)) AND (“pentaspline” OR “FARAPULSE (FP)” OR “FARAWAVE (FW)”) AND (“Complications” OR “hemolysis” OR “bleeding” OR “embolism” OR “injury” OR “stenosis” OR “adverse events”). All processes, including systematic search and study appraisal, were conducted per the guidelines of the Preferred Reporting Items for Systematic Reviews and Meta‐Analyses (PRISMA) statement [11].

### 2.2 Eligibility Criteria

Candidate articles were those reporting on technical, clinical, and laboratory outcomes among different PFA systems in patients with atrial fibrillation. Studies were considered eligible if they satisfied the following criteria: (1) original articles, (2) directly compare different PFA systems in terms of baseline and follow‐up outcomes, (3) include a minimum of 30 participants per compared group, (4) contain at least one reported metric of outcomes (e.g., technical, clinical, or laboratory), and (5) published in the English language.

Furthermore, manual exploration of the bibliographies of selected articles and retrieved systematic reviews was performed to find eligible studies that may have been missed by the initial screening processes. Only cohort studies or randomized trials were included. Other original study types (e.g., case reports, case series, commentaries, qualitative studies), and secondary studies (e.g., narrative reviews) were excluded.

### 2.3 Data Screening and Extraction

Using the above‐mentioned inclusion criteria, articles were independently screened by two authors across two stages. Primary screening by title and abstract, followed by secondary screening through full‐text evaluations. Findings from both authors were cross matched for consistency while conflict was resolved by consensus. If no consensus was reached, the final decision was imparted to the most senior author. No automation tools were used at any stage of study selection.

The following study characteristics and outcomes were extracted from the final list of eligible articles: general characteristics (study identifiers, country of origin, year of publication, methodological design, number of participants in total and per PFA system group, age, and male‐to‐female ratio), technical characteristics (procedural time, left arterial dwelling time, fluoroscopy time, and follow‐up duration), clinical outcomes (major and minor adverse events and vascular access site complications), laboratory outcomes (red blood cell count, total blood hemoglobin level, haptoglobin level, lactate dehydrogenase (LDH), total bilirubin levels, and creatinine).

### 2.4 Risk of Bias Assessment

The Newcastle‐Ottawa Scale (NOS) was used to assess methodological quality and risk of bias across included study types [12]. The scale has a maximum of nine points across three domains: selection, comparability, and outcome/exposure. Evaluations were conducted independently by two authors and were cross‐matched for possible discrepancies, which, if found, were resolved by a senior researcher. The final evaluation is provided as (Table S1).

### 2.5 Strategies for Data Synthesis and Analysis

The meta‐analysis was conducted using R (version 4.3.3, R Core Team, Vienna, Austria) using the “meta” package. Effect sizes were pooled and expressed alongside their 95% confidence intervals (CI). Heterogeneity among effect sizes was evaluated using the *I* squared statistic. Definitions for heterogeneity were adapted from the Cochrane handbook (> 25% mild, 25%–50% moderate, > 50% severe). Due to the anticipated high I squared value (i.e., greater than 50%), a random effects model was utilized.

For meta‐analysis of proportions, outcome rates were calculated as the number of affected individuals divided by the total number of participants. Differences in proportions were expressed as odds ratios (OR) between different PFA systems. Proportions were pooled using a random‐effects Freeman–Tukey double‐arcsine (PFT) transformation (REML τ^2^; Hartung–Knapp), 0%/100% studies were kept without correction and estimates were inverse‐transformed.

For meta‐analysis of mean differences, outcomes were pooled as standardized mean differences with associated CIs. For studies reporting medians and interquartile ranges, the mean and standard deviation were estimated using the “estmeansd” and “metamedian” packages [13, 14]. Standardized mean differences were pooled using the Hedges and Olkin formula (REML τ^2^; Hartung–Knapp). Sensitivity analysis was conducted per publication year and sample size. Publication bias was evaluated by funnel plots and egger's test of asymmetry (when applicable) (Figures S1–S8). A two‐sided *p* value of less than 0.05 was considered statistically significant for all conducted analyses.

## 3 Results

### 3.1 Search Results and Study Characteristics

**FIGURE 1:**
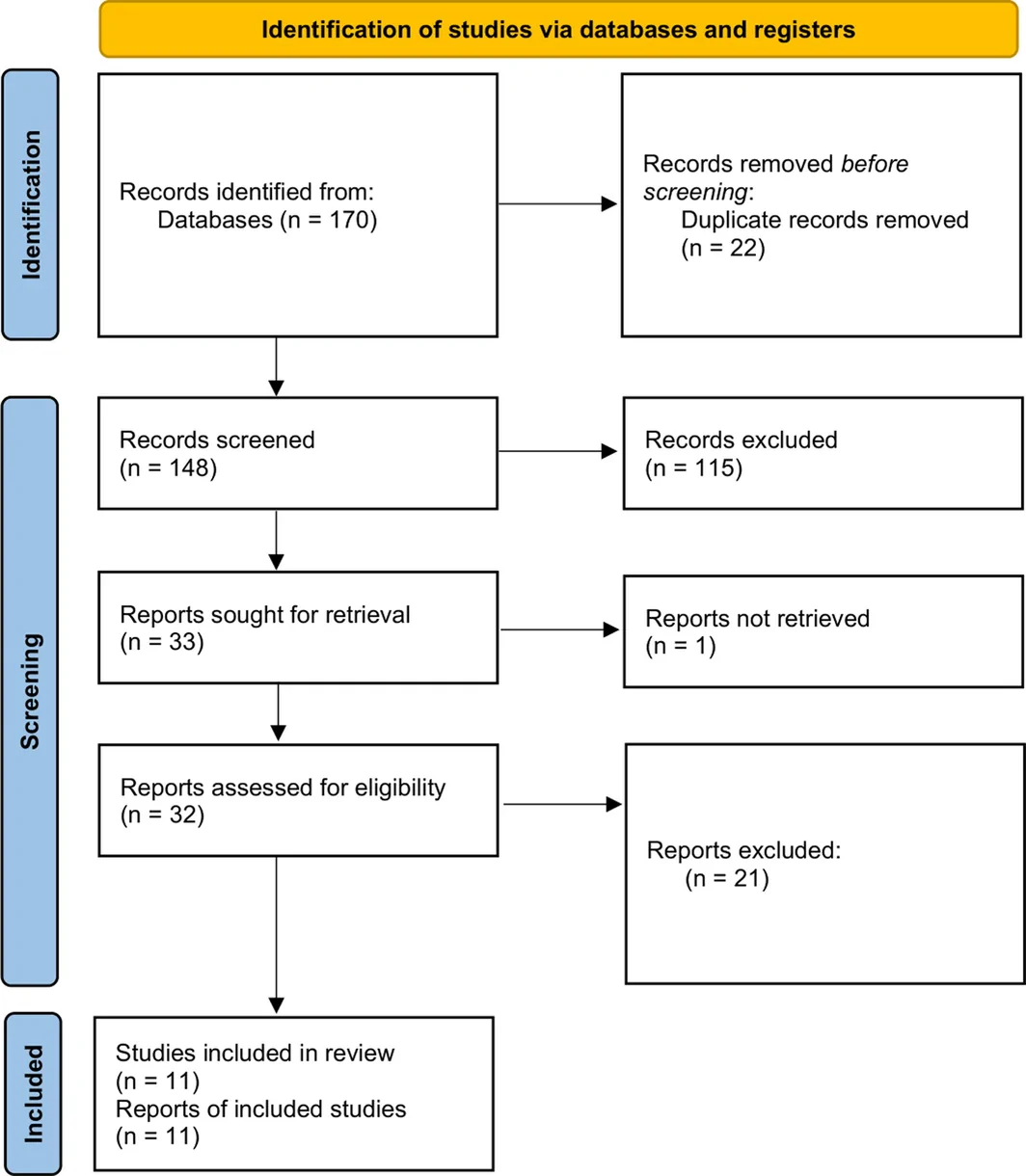
PRISMA chart for included studies.

Our database search identified 170 articles. After removing duplicates, 148 articles underwent primary screening. Of the screened articles, only 33 studies were subjected to a full‐text evaluation, after which 11 were included in the final qualitative analysis and 11 were included in the final quantitative synthesis (Figure 1). Although variable loop circular catheters (e.g., VARIPULSE) were included in the initial search, they were excluded from the final analysis due to insufficient data reporting. Among the studies included in the quantitative analysis, a total of 1517 individuals were examined. The pentaspline catheter pooled cohort was comprised of 674 individuals, while the circular catheter cohort was comprised of 841 individuals. All studies were published between 2025 and 2026. These studies originated from Japan (*n* = 5), Germany (*n* = 2), United States (*n* = 1), Canada (*n* = 1), Switzerland (*n* = 1), and only a single multi‐centric study. All included studies were observational cohorts and not randomized controlled trials. The characteristics of the included studies are presented in Table 1.

**TABLE 1:** Characteristics of included studies.

ID	Total participants	Number of participants (Circular)	Number of participants (Pentaspline)	Country of origin	Study design	Number of applications (Circular vs. Pentaspline)	Reported markers	Reported hemolysis markers	Reported markers of renal function	Follow‐up duration	Risk‐of‐bias score (NOS)
Abeln et al. [15]	402	175	227	Multi‐centric	Observational Cohort	33 vs. 34	Procedural parameters Safety outcomes	NA	NA	30 Days	8 (Low)
Yogarajah et al. [16]	60	30	30	Germany	Observational Cohort	35 vs. 32	Procedural parameters Safety outcomes	NA	NA	Not reported	8 (Low)
Zylla et al. [17]	80	40	40	Germany	Observational Cohort	NA	Procedural parameters Safety outcomes	NA	NA	8 weeks	8 (Low)
Kawamura et al. [18]	78	38	38	Japan	Observational Cohort	53 vs. 53	Procedural parameters Markers of hemolysis Markers of renal function	Total hemoglobin Plasma Free Hemoglobin LDH Bilirubin Haptoglobin	Creatinine	Not reported	8 (Low)
Kuraoka et al. [19]	105	66	39	Japan	Observational Cohort	57.3 vs. 46.4	Markers of hemolysis Markers of renal function	Total hemoglobin Plasma Free Hemoglobin LDH Bilirubin Haptoglobin	Creatinine; eGFR	Not reported	7 (Low)
Masuda et al. [20]	50	25	25	Japan	Observational Cohort	43 vs. 36	Procedural parameters Markers of hemolysis Markers of renal function	• Haptoglobin	Creatinine	Not reported	7 (Low)
Mountantonakis et al. [21]	183	108	75	USA	Observational Cohort	56.9 vs. 50.1	Procedural parameters Markers of hemolysis Markers of renal function	Total hemoglobin Plasma Free Hemoglobin LDH Bilirubin Haptoglobin	Creatinine; GFR	Not reported	7 (Low)
Ranganathan et al. [22]	224	209	15	Canada	Observational Cohort	NA	Markers of hemolysis Markers of renal function	Plasma Free Hemoglobin LDH Bilirubin Haptoglobin Reticulocyte count	Creatinine; eGFR	Not reported	6 (Moderate)
Miyazawa et al. [23]	105	62	43	Japan	Observational Cohort	39.8 vs. 45.3	Procedural parameters Markers of hemolysis Markers of renal function	Total hemoglobin LDH Bilirubin Haptoglobin	Creatinine; eGFR	Not reported	8 (Low)
Matsumoto et al. [24]	95	48	47	Japan	Observational Cohort	56 vs. 40	Procedural parameters Markers of hemolysis	LDH Bilirubin Haptoglobin LD isoenzyme 2	NA	Not reported	7 (Low)
Bruegger et al. [25]	135	40	95	Switzerland	Observational Cohort	34 vs. 34	• Markers of hemolysis	LDH Bilirubin Haptoglobin	NA	Not reported	Unknown

### 3.2 Technical Parameters

**FIGURE 2:**
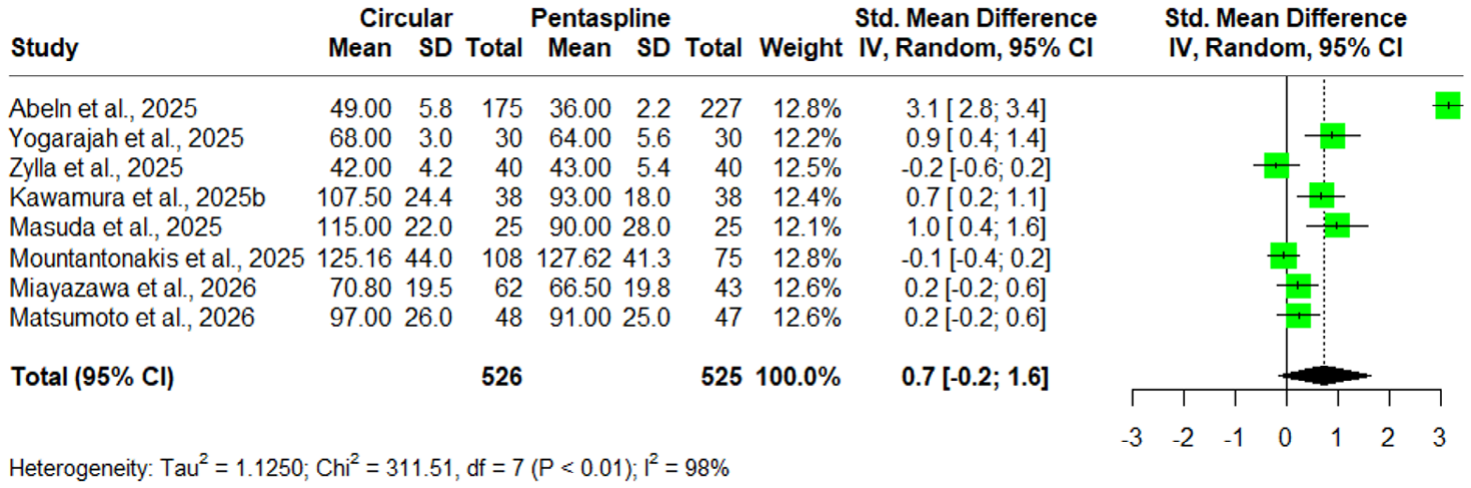
Forest plot of the pooled procedural time among included studies.

A total of eight studies reported procedural times for either the pentaspline or circular catheters [15, 16, 17, 18, 20, 21, 23, 24]. Mean procedural times for the circular catheter ablation ranged from 42 to 125 min, while procedural times ranged from 36 to 127 min in pentaspline‐directed therapy. The differences between the two PFA systems in terms of procedural time were statistically insignificant (SMD: 0.74; 95% CI: −0.16 to 1.63; *p* = 0.093) (Figure 2). Heterogeneity was extremely high and significant at an *I* ^2^ value of 97.8% (*p* < 0.001).

**FIGURE 3:**
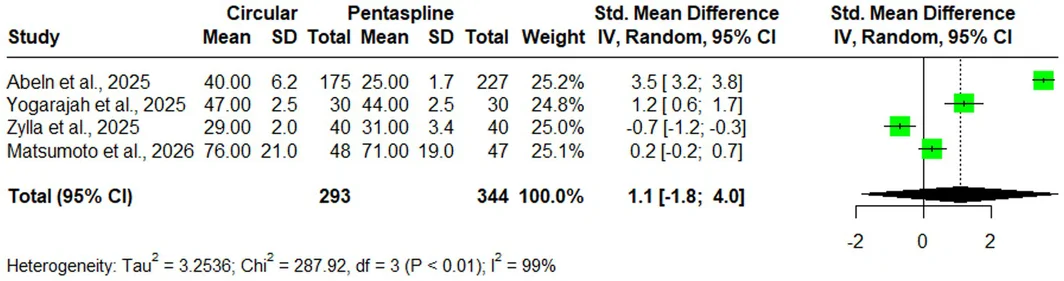
Forest plot of the pooled left atrial dwelling time among included studies.

Only four studies reported on left atrial dwelling time [15, 16, 17, 24]. For the circular catheter, mean dwelling times ranged from 29 to 76 min, while ranging from 25 to 71 min for the pentaspline system. There were no significant differences in mean arterial dwelling time between the two systems (SMD: 1.07; 95% CI: −1.82 to 3.95; *p* = 0.324) (Figure 3). Heterogeneity was extremely high and significant at an *I* ^2^ value of 99.0% (*p* < 0.001).

**FIGURE 4:**
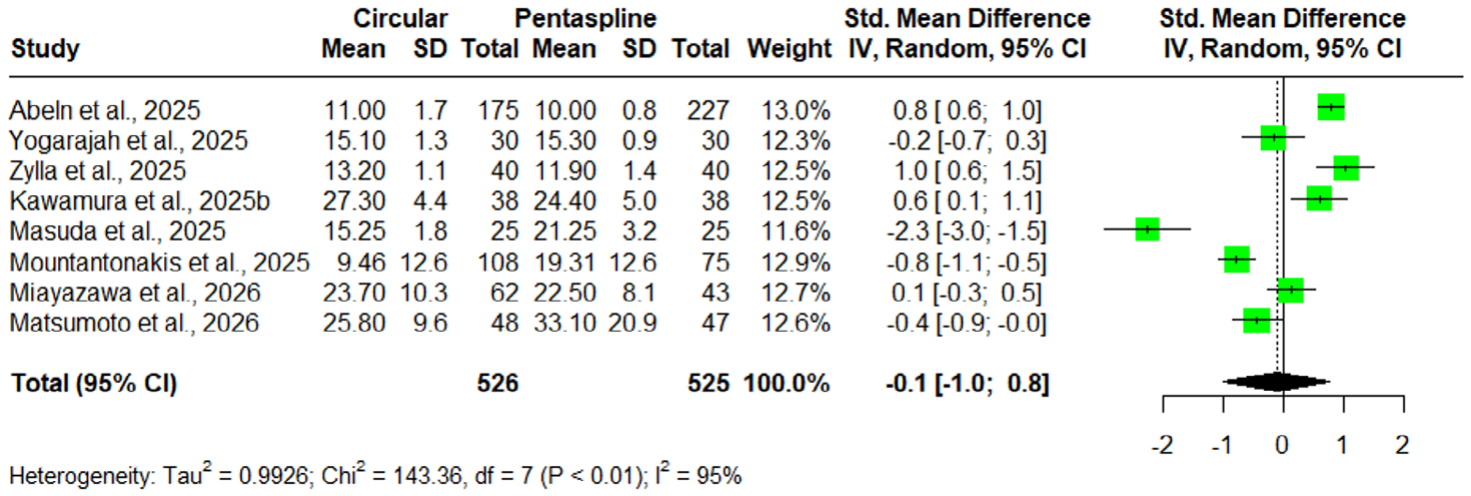
Forest plot of the pooled fluoroscopy time among included studies.

Fluoroscopy time was reported by eight studies [15, 16, 17, 18, 20, 21, 23, 24]. For the cohort treated with circular catheters, fluoroscopy times ranged from 9.5 to 27.3 min. Fluoroscopy time ranged from 10 to 33.1 min for the pentaspline cohorts. The mean difference between circular and pentaspline PFA systems in terms of fluoroscopy time was statistically insignificant (SMD: −0.12; 95% CI: −0.99 to 0.75; *p* = 0.759) (Figure 4). Heterogeneity was extremely high and significant at an *I* ^2^ value of 95.1% (*p* < 0.001). Subgroup analysis by the number of applications did not reduce the extreme heterogeneity in procedural, arterial dwelling, or fluoroscopy times (Figures S9–S11).

### 3.3 Biomarkers of Hemolysis

**FIGURE 5:**
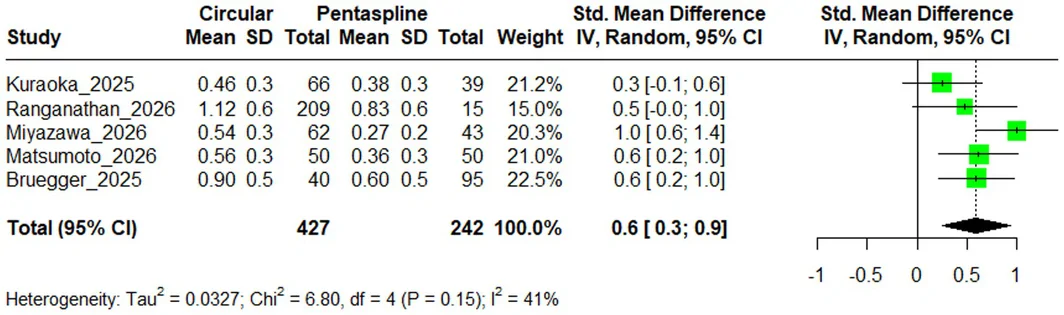
Forest plot of the post‐procedural haptoglobin between circular and pentaspline cohorts.

In terms of the impact of PFA catheter on haptoglobin changes, the majority of studies demonstrated that PFA ablation, irrespective of system, is associated with decreased post‐procedural haptoglobin at 24 h. Seven studies demonstrated that the magnitude of decrease in pre‐ to post‐procedure haptoglobin levels was significantly greater in pentaspline catheters compared to circular catheters [18, 19, 20, 22, 23, 24, 25]. On the other hand, one study found no significant differences in the magnitude of post‐procedural haptoglobin change [21]. In our meta‐analysis, a total of five studies included data regarding post‐procedural differences in haptoglobin levels between circular and pentaspline systems. Across 669 observations (PS: 427; FP: 242), there was a statistically significant difference between the two PFA systems in terms of post‐procedure haptoglobin levels (SMD: 0.59; 95% CI: 0.25–0.93; *p* = 0.008) (Figure 5). Among the included studies, heterogeneity was insignificant at *I* ^2^ of 41.2% (*p* = 0.1459).

**FIGURE 6:**
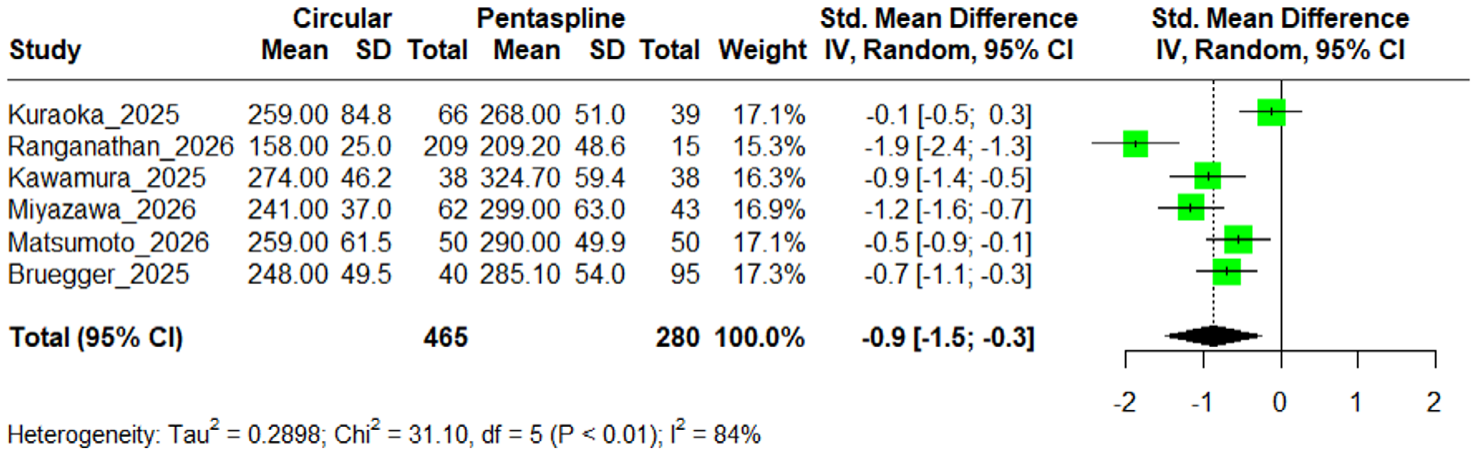
Forest plot of the post‐procedural LDH between circular and pentaspline cohorts.

A similar trend was observed for LDH among the included studies. Five studies reported higher magnitudes of LDH increases for pentaspline catheters compared to their circular counterparts [18, 19, 22, 23, 24, 25]. Only Mountantonakis et al. reported no differences in LDH levels between different PFA systems [21]. In this meta‐analysis, a total of six studies investigated post‐procedural changes in LDH levels between pentaspline and circular catheters. Across a total of 745 observations (PS: 465; FP: 280), there was a statistically significant difference in post‐procedural LDH between the two PFA systems (SMD: −0.87; 95% CI: −1.49 to −0.253; *p* = 0.015) (Figure 6). Heterogeneity was significantly high at an *I* ^2^ value of 83.9% (*p* < 0.001).

**FIGURE 7:**
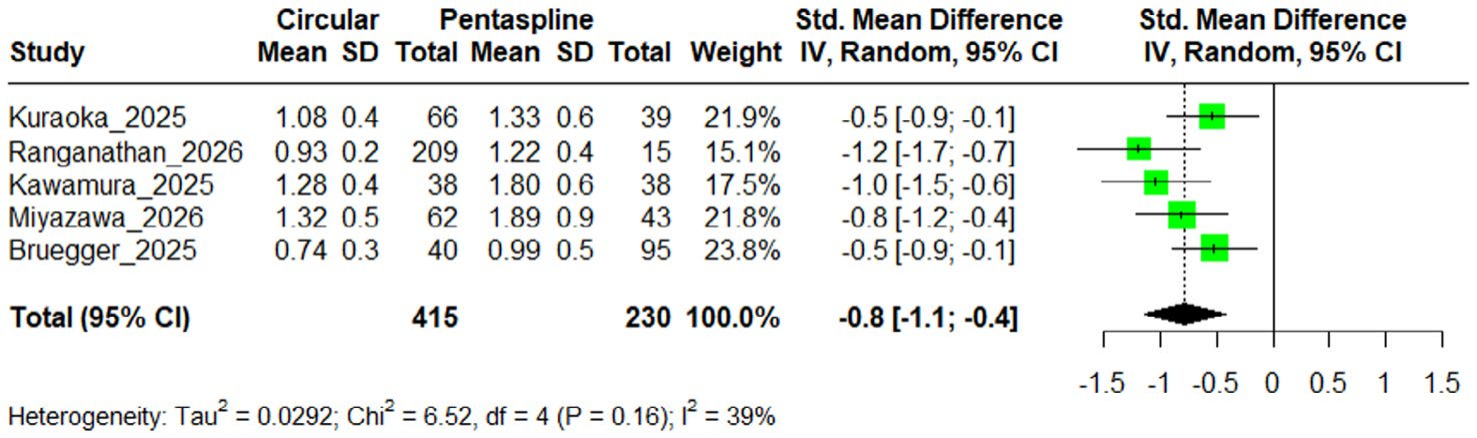
Forest plot of the post‐procedural bilirubin between circular and pentaspline cohorts.

The greater majority of studies demonstrated higher magnitudes of change in pre‐ to post‐procedure total bilirubin levels for the pentaspline system compared to circular catheters [18, 19, 23, 24, 25]. Conversely, two studies did not witness such statistical difference [21, 22]. The forest plot presented in Figure 7 demonstrated that a significant difference in post‐procedural bilirubin exists between pentaspline and circular catheters across a total of 645 observations (PS: 415; FP: 230) among five studies (SMD: −0.77; 95% CI: −1.13 to −0.42; *p* = 0.003). Heterogeneity was insignificant at an *I* ^2^ value of 38.7% (*p* = 0.163).

**FIGURE 8:**
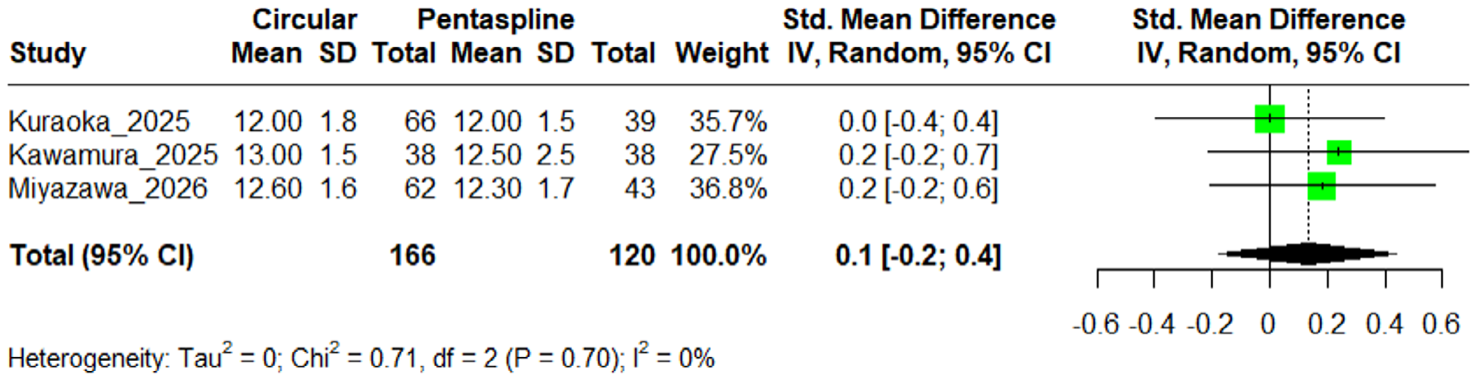
Forest plot of the post‐procedural hemoglobin between circular and pentaspline cohorts.

Kuraoka et al. demonstrated significant decreases in total hemoglobin 24 h after PFA procedures [19]. Moreover, there was no difference in the magnitude of total hemoglobin decrease between PFA systems. These findings were replicated by Miyazawa et al. and Kawamura et al. [18, 23]. Additionally, three studies showed that PFA ablation is associated with both significant increases in free plasma hemoglobin concentrations and a greater magnitude of free plasma hemoglobin increases in the pentaspline system compared to its circular counterpart [18, 21, 22]. In this meta‐analysis, among three studies harboring 286 observations (PS: 166; FP: 120), there were no statistically significant differences in post‐procedure total hemoglobin (SMD: 0.13; 95% CI: −0.17 to 0.44; *p* = 0.206) (Figure 8). Heterogeneity was insignificant at an *I* ^2^ value of 0.0% (*p* = 0.701).

On subgroup analysis by number of PFA applications, it appears that the number of PFA applications did not influence the heterogeneity of pooled markers of hemolysis. The forest plots depicting the aforementioned analysis are present in Figures S12–S16.

### 3.4 Predictors of Hemolysis

Only a handful of studies examined the risk factors associated with hemolysis. Ranganathan et al., demonstrated that ablation strategy (i.e., pulmonary vein isolation plus posterior wall isolation), higher lesion count, and catheter type were independent predictors of severe hemolysis [22]. On the other hand, Montantonakis et al., demonstrated that only catheter type was an independent predictor of hemolysis after adjustment for age, sex, GFR, creatinine, hemoglobin, and ablation strategy [21]. Finally, Miyazawa et al., showed that PFA application ratio and body mass index were independent predictors of decreased haptoglobin ratio [23]. The latter model was adjusted for age, biological sex, hemoglobin levels, eGFR, left atrial diameter, RFA energy, among others.

### 3.5 Markers of Renal Function

**FIGURE 9:**
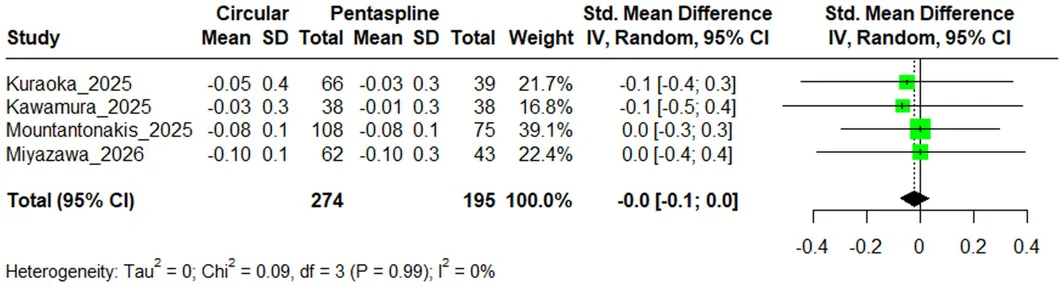
Forest plot of the pre/post‐procedural differences in creatinine between circular and pentaspline cohorts.

Of the included literature reporting on renal function (*n* = 7), all had observed no significant impact from PFA unto creatinine levels [18, 19, 20, 21, 22, 23]. In this meta‐analysis, a total of four studies investigated pre‐to‐post procedural changes in creatinine levels between pentaspline and circular catheters [18, 19, 21, 23]. Across a total of 469 observations in four studies, there were no significant differences in magnitude of creatinine change between the two PFA systems (SMD: −0.02; 95% CI: −0.07 to 0.030; *p* = 0.270) (Figure 9). Heterogeneity was insignificant at an *I* ^2^ value of 0.0% (*p* = 0.992).

This trend was replicated for studies investigating GFR or eGFR [21, 22]. Interestingly, a total of five studies reported on the number of observed AKI across their samples [18, 19, 20, 23, 24]. The majority of studies defined AKI based on the KDIGO criteria [18, 19, 23], one study based on the RIFLE classification [24], while one study failed to include their definition of AKI [20]. Overall, there was no evidence of AKI within three different cohorts. Conversely, three studies demonstrated AKI exclusively in patients with pentaspline catheters. While Masuda et al. reported a single case [20], Miyazawa et al. and Matsumoto et al. reported two within each of their respective cohorts [23, 24].

### 3.6 Non‐Renal Safety Profile

Only four studies had described the peri/post procedural complications associated with the circular and pentaspline PFA systems [15, 16, 17, 24]. In their study of 175 PulseSelect and 227 FARAPULSE cases, Abeln et al. documented a total of seven complications among patients treated with PulseSelect and 63 complications among those treated with FARAPULSE over a 30‐day follow‐up period [15]. Complications for the PulseSelect group included two thromboembolic events, two vascular access site complications, two instances of minor bleeding, and one stroke. Conversely, the FARAPULSE had 28 cases of vascular access site complications, 27 cases of minor bleeding, five cases of vagal reactions during ablation, and one case of thromboembolic events, transient ischemic attacks, and major bleeding. Yogarajah et al. studied 30 cases of PulseSelect and FARAPULSE patients and documented a single case of vascular access site complications in the PulseSelect cohort [16]. Across 40 cases treated with PulseSelect and FARAPULSE, respectively, Zylla et al. reported five complications (one major; four minor) in the PulseSelect cohort and three complications (two major; one minor) in the FARAPULSE cohort [17]. Complications for the PulseSelect group include a single case of cardiac tamponade, two cases of air embolism, one case of minor bleeding, and a single diagnosed arteriovenous fistula. On the other hand, the FARAPULSE cohort had two cases of transient ischemic attacks and a single diagnosed arteriovenous fistula. Cardiac tamponade in PulseSelect was reported in a single case by Matsumoto et al. [24].

## 4 Discussion

### 4.1 Summary of Findings

Our meta‐analysis found that the extent of hemolysis is more pronounced with pentaspline catheters in comparison to circular catheters. This was evident clinically in terms of a more pronounced consumption in haptoglobin and greater elevation in LDH and total bilirubin. Nevertheless, there were no statistical differences in terms of total hemoglobin between the two PFA systems. Furthermore, no differences in markers of renal functions were detected between pentaspline and circular catheters. However, the incidence of AKI among the pentaspline cohort was more pronounced at five cases across three different studies. Finally, more peri/post‐procedural complications were documented for the pentaspline system in comparison with circular catheters. It should be noted that for all conducted pooling, heterogeneity was substantial, indicating a difference in methodological quality, data collection procedures, and confidence in reporting.

### 4.2 Pathophysiology of PFA‐Induced Hemolytic Damage

Despite being relatively resistant to mechanical force and shearing stress, erythrocytes remain susceptible to electrical field damage. Kinosita et al. demonstrated that erythrocytes' hemolysis is driven by disturbances in membrane potential which allows ions' leakage and subsequent osmotic imbalance that eventually leads to cellular rupture and subsequent death [26]. A recent in vitro study calculated the electrical energy for hemolysis to occur, which was estimated at a minimum of 1000 V/cm [7]. Interestingly, while a current of 750 V/cm is safe on erythrocytes and functional in inducing cardiac myocytes' apoptosis, the effectiveness of the electrical current is logarithmic, with higher energies being associated with significantly higher percentage of myocytes' apoptosis. Therefore, the technology associated with PFA requires a delicate balance between achieving pulmonary vein isolation and minimizing hemolysis.

At a glance, the higher voltage of the pentaspline catheter could explain its significantly higher hemolytic complications compared to circular catheters (2000 V vs. 1500 V) [18]. However, it appears that regardless of the amount of energy used, the proximity of the ablation catheter to the target tissue puts erythrocytes at an increased risk of supra‐physiological electrical current and hemolysis [27]. This phenomenon could explain the differences in the degree of hemolysis observed among different PFA systems; as the gaps between the splines allow the electric field to interact more directly with the intervening, central blood flow in between those splines, a phenomenon that is not possible with the continuous, ring design of circular catheters.

### 4.3 The Impact of Application Number

It is confirmed that the relationship between number of PFA applications and hemolysis is rather linear [21, 22]. Bruss et al. suggest that it is dose‐dependent [28]. Therefore, it is suggested to tailor number of PFA applications to clinical needs [4, 19, 29]. It is a given that higher number of applications is needed to reduce AF recurrence [30]; thus, circular catheters might be suitable for ablations of extended duration. Kuraoka et al. further demonstrate that there is indifference of circular catheters to number of applications [19]. In their study, the pentaspline technique was limited to a maximum of 70 applications compared to no limits for the circular catheter, of which results showed comparable hemolysis damage.

### 4.4 Markers of Hemolysis: What to Rely on

Optimally, free plasma hemoglobin and RBC microparticles are considered the most specific, sensitive, and accurate markers of ongoing intravascular hemolysis [31]. In addition to their diagnostic efficacy, these markers parallel the progress of hemolysis in a continuum. A number of studies found positive correlations between PFA number and the aforementioned markers, further cementing their heightened sensitivity towards PFA‐related hemolysis [4]. In fact, Nies et al. demonstrated a dose‐dependent relationship between free plasma hemoglobin and number of PFA applications [32]. However, free plasma hemoglobin and RBC microparticles are limited by their limited availability in clinical settings, and in the case of RBC microparticles, lack standardization protocols for measurement or analysis [28]. Additionally, due to their rapid clearance, reliance on free plasma hemoglobin or RBC microparticles 24 h post‐ablation may underestimate hemolysis.

Haptoglobin presents itself as an alternative marker for detection of mild hemolysis if the earlier markers were not available [19]. In vitro studies demonstrate almost an inverse linear correlation between free hemoglobin and haptoglobin levels, supporting its use to detect hemolysis [33]. Matsumoto et al. observed temporal changes in haptoglobin as far as 24 h post ablation; thus, providing a wider window for hemolysis detection [24]. Interestingly, a number studies showcased the predictive capacity of haptoglobin with regards to the incidence of renal injury. For example, differences in levels of baseline haptoglobin were associated with varying incidence of early renal injury [34, 35]. Tanaka et al. suggest that the variance in baseline haptoglobin levels presents a facet of individual vulnerability that may influence renal outcomes, in which higher levels of haptoglobin act as a buffer against hemolytic stress [34, 36]. Suga et al. further expand on the buffering role of baseline haptoglobin levels by indicating that different haptoglobin phenotypes are associated with renal injury by influencing levels of haptoglobin circulation [35]. Specifically, patients with haptoglobin phenotype 2‐2 were more likely to exhibit the lowest levels of haptoglobin, and therefore, the highest incidence of post‐operative AKI [37]. In summary, baseline haptoglobin could be utilized as a proxy marker that could help identify patients with increased susceptibility to renal stress and/or complications.

However, if the capacity of haptoglobin is exhausted, hemolysis could be underestimated as it will continue without reflection in its levels. The other conventional markers of hemolysis, such as LDH and bilirubin levels, are unreliable within the context of post‐ablation hemolysis. Despite their availability and ease of interpretation, they are easily affected by other pathophysiological processes (e.g., inflammation, myocardial damage). In the case of LDH, increased levels most likely reflect a composite of LDH released by RBC during hemolysis and cardiac cells during ablation [4]. This lack of specificity limits the quantification of hemolysis severity in either direction. This also limits comparisons between different PFA modalities unless pre‐procedural LDH is matched and statistically balanced. Nonetheless, the increase in LDH is only complementary to decreased haptoglobin, significantly improving the sensitivity of the latter to hemolysis [27, 31].

### 4.5 Methodological Critique and Recommendations

As evidenced by the substantial heterogeneity across the included studies, future studies investigating hemolysis, renal function, and safety of different PFA systems must take into consideration the following considerations. For optimal standardization, future researchers should strive to adopt a unified data collection standard, particularly for post‐procedural markers of hemolysis. Reporting laboratory markers of hemolysis pre‐procedure, immediately post‐procedure, and 24‐h post‐procedure are the most common standards which allow for direct comparability despite the latter not consistently being able to capture the peak of hemolytic changes. For indirect measures of hemolysis, pre‐procedural matching is of utmost importance as these markers are often influenced by clinical and intervention characteristics. Immediate post‐procedural measurements are crucial only when free plasma hemoglobin or RBC microparticles are utilized as the primary markers for hemolysis detection. These markers lose predictive power during the 24‐post procedure period, after which others, such as haptoglobin, become more reliable over a lengthy temporal frame.

Different studies may report unique sets of hemolytic markers and, at times, extremely obscure, which may not be commonly used in all institutions or routine clinical practice. We recommend reporting free plasma hemoglobin, haptoglobin, and LDH irrespective of other included markers. If available, free plasma hemoglobin is the most accurate and reliable marker of hemolysis during the procedure and immediately post‐procedure. The combination of haptoglobin and LDH was shown to be consistently predictive of renal injury [27, 31, 38]. While hemoglobinuria is an important sign of clinical hemolysis, it typically occurs after depletion of haptoglobin, further cementing the status of haptoglobin levels as a reasonable surrogate marker of early hemolysis [39]. Similarly, creatinine, GFR, and urine hemoglobin should be consistently reported for assessment of renal function. These markers are also useful to monitor hemolysis post the 24‐post procedure period, after which haptoglobin levels may be depleted. Studies should strive to adopt similar cut‐off points for detection of hemolysis or renal failure. However, ROC analysis of proposed cut‐offs is also encouraged with or without external validation.

Studies should strive to adjust not only for the number of PFA applications, especially in the pentaspline cohorts, but also for any baseline characteristic that could affect the degree of hemolysis. This is particularly relevant for markers of cardiac injury, as those were found to be mediators of fluctuations in presented markers of hemolysis [38]. Additionally, only a handful of included studies provided an appropriate timeframe for follow‐up of outcomes. In fact, most studies failed to examine hemolysis beyond the 24‐h period after the procedure. Providing substantial follow‐up is pertinent for the assessment of safety parameters and chronic renal function.

To note, the small sample sizes in an array of different included studies could lead to reduced statistical power; thus, an inherent inability to detect differences between PFA systems or different time‐points for the same system. A data‐driven sample size calculation procedure per used statistical test is recommended. Finally, studies should, at minimum, report their procedural strategies in sufficient detail. This includes experience of operators with ablation systems, hydration protocols, and indication for PFA usage. The latter is especially important; as not all PFA procedures were utilized for primary treatment.

Overall, it appears that catheter design, differences in institutional protocols, in addition to individual vulnerabilities may influence the incidence of hemolysis and/or significant renal dysfunction in patients undergoing PFA ablation. Therefore, in an attempt to create a risk‐stratification model for renal injury, future research must examine PFA system differences within the context of the hemolytic buffering capacity of patients. This particularly concerns baseline levels of haptoglobin or theoretically hemopexin [40]. However, multiple critical questions remained unanswered. While our pooling of data shows a straightforward trend between worsening of certain hemolytic markers and PFA systems, the precise threshold at which transient hemolysis crosses into AKI remains undefined. In other words, the hard limit of the anti‐hemolysis buffering capacity of susceptible patients remains unknown. Other factors not considered across the literature include host genetic susceptibility (e.g., haptoglobin phenotypes), volume of PFA energy delivered, and ablation strategy (i.e., additional ablation targets).

## 5 Limitations

Our findings are preliminary and should be interpreted within the context of a number of limitations. Not all markers of hemolysis or complications satisfied the criteria for pooling. In some cases, pooling was barely doable. This reflects the heterogeneity in terms of reporting among included studies. Due to the limited number of studies, including the variable loop circular catheters into the pooling was not possible nor was it possible to conduct a meta‐regression to examine the influence of PFA applications or sample size on the pooled effects. Furthermore, the sample size for a number of studies was modest, which could have introduced a small‐study effect into our pooled estimates. On sub‐group analysis, the limited number of studies could have diluted the differences in hemolysis markers across different PFA systems. It should be noted that due to differences in reporting continuous variables (means vs. medians), conversions could have introduced an arithmetic bias as means cannot be reliably inferred with 100% accuracy from medians due to the different mathematical properties of each metric. The high level of methodological heterogeneity across some of the included markers of hemolysis warrants caution at interpretation. While procedural characteristics and protocols were similar in between studies, unmeasured sources of heterogeneity, such as baseline differences in haptoglobin or haptoglobin phenotypes, were not controlled for. The lack of standardized genotyping across the literature remains a key limitation that future prospective studies must address. Finally, only three databases were examined for relevant literature, which could have introduced a risk of selection bias.

## 6 Conclusion

Our meta‐analysis demonstrated that pentaspline systems were associated with more pronounced markers of hemolysis. Qualitatively, complication rates including AKI were reported more often in these systems. Nonetheless, clinically significant renal dysfunction remained uncommon and evidence regarding clinical consequences remains limited. Regardless of the system used, we noticed heterogeneity in reporting the findings of the individualized studies that need to be addressed by researchers; we encourage the investigators of future projects investigating this phenomenon to adopt reporting the same hemolytic markers, report their findings using the same units and measure of central tendency, define a specific time point for post procedural blood withdrawal and extend their follow up period.

## Author Contributions

Conceptualization: Abdalrahman Al‐Slaimieh, Abdallah Al‐Ani. Supervision: Mohammad Hajjiri. Data curation: Abdalrahman Al‐Slaimieh, Abdallah Al‐Ani, Rand Al‐Huneidy. Formal analysis: Abdalrahman Al‐Slaimieh, Abdallah Al‐Ani. Methodology: Abdalrahman Al‐Slaimieh, Abdallah Al‐Ani. Writing – original draft: Abdalrahman Al‐Slaimieh, Rand Al‐Huneidy, Ibrahim Ghayada, Mohammad Shahin, Khaled Abukhalaf, Waleed Alomari, Khaled Marwa, Saeed Abdel Kamel, Yahya Almazaraa, Abdallah Al‐Ani, Mohammad Hajjiri. Writing – review and editing: Abdalrahman Al‐Slaimieh, Abdallah Al‐Ani, Rand Al‐Huneidy, Mohammad Hajjiri.

## Funding

The authors have nothing to report.

## Ethics Statement

The authors have nothing to report.

## Consent

The authors have nothing to report.

## Conflicts of Interest

The authors declare no conflicts of interest.

## Supporting information


**Table S1:** Procedural protocols and characteristics of included studies.
**Figure S1:**: Funnel plot of the standardized mean differences in procedure time versus the standard error for each of the included studies.
**Figure S2:**: Funnel plot of the standardized mean differences in left arterial dwelling time versus the standard error for each of the included studies.
**Figure S3:**: Funnel plot of the standardized mean differences in fluoroscopy time versus the standard error for each of the included studies.
**Figure S4:**: Funnel plot of the standardized mean differences in post‐procedure haptoglobin levels versus the standard error for each of the included studies.
**Figure S5:**: Funnel plot of the standardized mean differences in post‐procedural LDH levels versus the standard error for each of the included studies.
**Figure S6:**: Funnel plot of the standardized mean differences in post‐procedural bilirubin levels versus the standard error for each of the included studies.
**Figure S7:**: Funnel plot of the standardized mean differences in post‐procedural total hemoglobin levels versus the standard error for each of the included studies.
**Figure S8:**: Funnel plot of the standardized mean differences in post‐procedural creatinine levels versus the standard error for each of the included studies.
**Figure S9:**: Subgroup analysis of standardized mean differences in procedural time per number of PFA applications.
**Figure S10:**: Subgroup analysis of standardized mean differences in left arterial dwelling time per number of PFA applications.
**Figure S11:**: Subgroup analysis of standardized mean differences in fluoroscopy time per number of PFA applications.
**Figure S12:**: Subgroup analysis of standardized mean differences in post‐procedural haptoglobin levels per number of PFA applications.
**Figure S13:**: Subgroup analysis of standardized mean differences in post‐procedural LDH levels per number of PFA applications.
**Figure S14:**: Subgroup analysis of standardized mean differences in post‐procedural total bilirubin levels per number of PFA applications.
**Figure S15:**: Subgroup analysis of standardized mean differences in post‐procedural total hemoglobin levels per number of PFA applications.
**Figure S16:**: Subgroup analysis of standardized mean differences in post‐procedural creatinine levels per number of PFA applications.

## Data Availability

The data that support the findings of this study are available from the corresponding author upon reasonable request.
