# Supplementary material for: Evaluation of Hemolysis Risk, Renal Function, and Safety Among Two Pulsed Field Ablation Systems: A Systematic Review and Meta‐Analysis
Source: J Arrhythm. 2026 Aug 31;42(5):e70450. doi: 10.1002/joa3.70450 (PMC13528620; doi:10.1002/joa3.70450)
Supplement: Supplementary file 1 [file JOA3-42-e70450-s001.docx]

**Evaluation of hemolysis risk, renal function, and safety among two pulsed field ablation systems: a systematic review and meta-analysis**

**Supplementary Materials**

**Supplementary Table 1**: Procedural protocols and characteristics of included studies

| **ID** | **Operator Experience** | **Procedural Workflow** | **Catheter Size** | **Hydration Protocol** | **Voltage Characteristics** | **Anti-Coagulation Protocol** | **ACT** |
| --- | --- | --- | --- | --- | --- | --- | --- |
| Abeln et al., 2025 | Not reported | • Fluoroscopy without 3D guidance | • Circular 9F • Pentaspline 12F | Not reported | • Circular 1400 - 1500 V • Pentaspline 2000 V | • Uninterrupted OAC | 300 |
| Yogarajah et al., 2025 | Not reported | • TEE • TTE • Fluoroscopy without 3D guidance | • Circular 9F • Pentaspline 12F | Not reported | Not reported | • Oral anticoagulants paused 12 hours prior to procedure • Vitamin K antagonists continued at (INR: 2.0 - 3.0) • Oral anticoagulants were resumed 3 hours after procedure and continued for at least 2 months | Not reported |
| Zylla et al., 2025 | Not reported | • TEE • Fluoroscopy with 3D guidance | • Circular 10F • Pentaspline 12F | Not reported | Not reported | • Oral anticoagulants paused 12 hours prior to procedure • Vitamin K antagonists continued at (INR: 2.0 - 2.5) | 300 - 400 |
| Kawamura et al., 2025 | Not reported | • TEE | Not reported | 1000ml (IV) | • Circular 1500 V • Pentaspline 2000 V | • Uninterrupted OAC | 300 - 400 |
| Kuraoka et al., 2025 | Not reported | • Fluoroscopy with 3D guidance | Not reported | 1ml/kg/hr | Not reported | Unknown | > 350 |
| Masuda et al., 2025 | Not reported | • Intra-Cardiac Echo • Fluoroscopy with 3D guidance | • Circular 10F • Pentaspline 13F | Not reported | Not reported | • Uninterrupted OAC | 350 |
| Mountantonakis et al., 2025 | Not reported | • Intra-Cardiac Echo • Fluoroscopy with 3D guidance | Not reported | No hydration | Not reported | • Uninterrupted OAC | > 350 |
| Ranganathan et al., 2025 | Not reported | • TEE • Intra-Cardiac Echo | Not reported | No hydration | • Circular 1400 - 1500 V • Pentaspline 1800 - 2000 V | • Uninterrupted OAC | > 300 |
| Miyazawa et al., 2026 | Not reported | • TEE • Fluoroscopy with 3D guidance | Not reported | 1000ml (IV) | • Circular 1500 V • Pentaspline 2000 V | • Uninterrupted OAC | 300 - 400 |
| Matsumoto et al., 2026 | Not reported | • Fluoroscopy with 3D guidance | Not reported | Not reported | Not reported | Unknown | >350 |
| Bruegger et al., 2026 | Not reported | Not reported | Not reported | Not reported | Not reported | Not reported | Not reported |

*TEE, Trans-esophageal echo; TTE, Trans-thoracic echo; ACT, activated clotting time

**Supplementary Figure 1**: Funnel plot of the standardized mean differences in procedure time versus the standard error for each of the included studies.

**Supplementary Figure 2**: Funnel plot of the standardized mean differences in left arterial dwelling time versus the standard error for each of the included studies.

**Supplementary Figure 3**: Funnel plot of the standardized mean differences in fluoroscopy time versus the standard error for each of the included studies.

**Supplementary Figure 4**: Funnel plot of the standardized mean differences in post-procedure haptoglobin levels versus the standard error for each of the included studies.

**Supplementary Figure 5**: Funnel plot of the standardized mean differences in post-procedural LDH levels versus the standard error for each of the included studies.

**Supplementary Figure 6**: Funnel plot of the standardized mean differences in post-procedural bilirubin levels versus the standard error for each of the included studies.

**Supplementary Figure 7**: Funnel plot of the standardized mean differences in post-procedural total hemoglobin levels versus the standard error for each of the included studies.

**Supplementary Figure 8**: Funnel plot of the standardized mean differences in post-procedural creatinine levels versus the standard error for each of the included studies.

**Supplementary Figure 9**: Subgroup analysis of standardized mean differences in procedural time per number of PFA applications

**Supplementary Figure 10**: Subgroup analysis of standardized mean differences in left arterial dwelling time per number of PFA applications

**Supplementary Figure 11**: Subgroup analysis of standardized mean differences in fluoroscopy time per number of PFA applications

**Supplementary Figure 12**: Subgroup analysis of standardized mean differences in post-procedural haptoglobin levels per number of PFA applications

**Supplementary Figure 13**: Subgroup analysis of standardized mean differences in post-procedural LDH levels per number of PFA applications

**Supplementary Figure 14**: Subgroup analysis of standardized mean differences in post-procedural total bilirubin levels per number of PFA applications

**Supplementary Figure 15**: Subgroup analysis of standardized mean differences in post-procedural total hemoglobin levels per number of PFA applications

**Supplementary Figure 16**: Subgroup analysis of standardized mean differences in post-procedural creatinine levels per number of PFA applications
